# Production and utilization of a high-density oligonucleotide microarray in channel catfish, *Ictalurus punctatus*

**DOI:** 10.1186/1471-2164-7-134

**Published:** 2006-06-01

**Authors:** Robert W Li, Geoffrey C Waldbieser

**Affiliations:** 1 USDA, Agricultural Research Service, Catfish Genetics Research Unit, Stoneville, MS 38776, USA; 2 USDA, Agricultural Research Service, Bovine Functional Genomics Laboratory, Beltsville, MD 20705, USA

## Abstract

**Background:**

Functional analysis of the catfish genome will be useful for the identification of genes controlling traits of economic importance, especially innate disease resistance. However, this species lacks a platform for global gene expression profiling, so we designed a first generation high-density oligonucleotide microarray platform based on channel catfish EST sequences. This platform was used to profile gene expression in catfish spleens 2 h, 4 h, 8 h and 24 h after injection of lipopolysaccharide (LPS).

**Results:**

In the spleen samples, 138 genes were significantly induced or repressed greater than 2-fold by LPS treatment. Real-time RT-PCR was used to verify the microarray results for nine selected genes representing different expression levels. The results from real-time RT-PCR were positively correlated (R^2 ^= 0.87) with the results from the microarray.

**Conclusion:**

The first generation channel catfish microarray provided several candidate genes useful for further evaluation of immune response mechanisms in this species. This research will help us to better understand recognition of LPS by host cells and the LPS-signalling pathway in fish.

## Background

The channel catfish, *Ictalurus punctatus*, is native to North America and is from the order Siluriformes (superorder Ostariophysi) which is considered more primitive among teleosts [1,2]. Commercial production of catfish as dietary protein is the leading industry of North American aquaculture, with more than 600 million pounds of catfish produced annually in the United States [3]. One of the most significant factors limiting production is loss to disease, and one of our goals is to improve non-specific immunity through selective breeding. The channel catfish immune system is the best characterized for any fish species, and it is the only fish species where clonal functionally distinct lymphocyte lines can be readily established [4]. However, there is a need to better understand the physiological and immunological pathways controlling host-pathogen interactions *in vivo*.

Improvement of disease resistance in catfish populations depends on an understanding of the genetic control of immune-related pathways, response to pathogens, and correlations with other production traits. Recent developments in genomic technology, particularly high-throughput cDNA sequencing and development of expressed sequence microarrays, have made possible the profiling of global gene expression in experimental fish tissues.

Microarray experiments have been utilized to determine regulation of gene expression during developmental and adaptive processes in several fish species. Using this technology, groups of coordinately expressed genes were identified in zebrafish embryos during development [5,6]. Microarrays were used to identify changes in gene expression patterns in zebrafish and goby exposed to differing levels of available oxygen [7,8], in common carp exposed to cold temperature [9], and in rainbow trout exposed to handling stress [10].

Recently, expression microarrays have been used to identify genes involved in host-pathogen relationships. Experimental infection of cells *in vitro *revealed differential gene expression in flounder kidney cells [11], and in rainbow trout monocytes and macrophages [12]. Transcriptional analysis was performed on kidney cells after experimental *in vivo *injection of flounder with a DNA vector expressing the viral hemorrhagic septicemia virus G protein [13], in Atlantic salmon macrophages and anterior kidney tissues after injection of fish with *P. salmonis *[14], on Atlantic salmon liver, spleen, and anterior kidney tissues after cohabitation with *A. salmonicida*-infected fish [15], and from whole zebrafish after infection with *M. marinum *[16]. By measuring changes in gene expression after pathogen challenge, researchers may identify gene expression fingerprints that provide clues to molecular pathways involved in pathogen neutralization and/or removal, identify candidate genes controlling pathogen-specific immunity, and identify heritable differences in gene expression levels that correlate with disease resistance/susceptibility. This data would help in the formulation of a selection index to identify broodstock with superior genetic potential for resistance to disease.

While microarray-based transcriptional analysis is a useful tool for functional genomics in fish, to date there has been no microarray platform available for catfish species. Thus, we developed a catfish microarray utilizing existing data from channel catfish expressed sequences obtained in cDNA cloning experiments from several tissues (reviewed in [17][18]). These sequences have been clustered and annotated in the Catfish Gene Index [19]. The catfish microarray was used to measure differential gene expression in the spleen of lipopolysaccharide-injected and sham-injected catfish.

## Results

A high density oligonucleotide microarray was produced that contained 18,989 catfish expressed sequences, each represented by ten perfectly matched and ten mismatched 24-mer oligonucleotides (GEO Database Accession GSE3261). This microarray contained 8057 sequences that matched an annotated sequence in GenBank. Discounting 875 that matched transposons or sequences that were labelled as unknown, unnamed, or hypothetical, there were 7182 annotated sequences (38%) on the microarray. This microarray was used to demonstrate differences in global gene expression in the spleen of LPS-treated catfish. Using Significance Analysis of Microarrays (SAM), we identified 138 sequences that were differentially expressed after LPS treatment [see Additional file 1 and Additional file 2].

There were 64 genes up-regulated by LPS exposure of which only 26 were annotated based on sequence similarity [see Additional file 1]. Among these were cytokines and chemokines, such as IL-1β, CCL4, a small CXC/IL 8-like chemokine, and chondromodulin II. Transcriptional factors such as NF-κB p100 subunit and NF-κB inhibitor alpha-like proteins A and B, interferon regulatory factor I, and AP-1 were also upregulated at least 2-fold. Expression of Toll-like receptor 5 was upregulated at 2–4 h post-exposure. Within the experimental timeframe, almost all induced genes were induced by 4 h post-exposure.

There were 74 genes that were significantly down-regulated in response to LPS, of which only 29 were annotated based on sequence similarity [see Additional file 2]. Among these genes were the immunoglobulin light and heavy chains, MHC class II antigens, invariant chain-like protein 2, an NK lysin-like protein, complement C3, hemoglobin alpha, and a CXC chemokine receptor.

**Figure 1:**
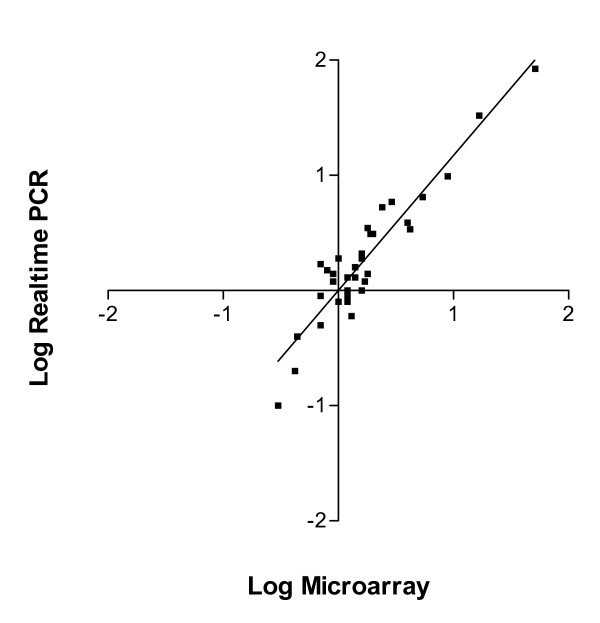
**Correlation between levels of gene expression measured by microarray and real-time RT-PCR**. The correlation (R^2 ^= 0.87) was calculated using log-transformed values of the fold change obtained for the nine selected genes. Four fish were used per treatment/time combination.

In the present research, we selected nine expressed sequences representing varying levels of expression to verify the microarray results using real-time RT-PCR [see Additional file 3]. Linear regression of log transformed expression data demonstrated a strong positive correlation (R^2 ^= 0.87) between the two technologies (Fig. 1). The microarray results for TNFα failed to pass our quality control criteria, thus no expression ratios were reported (detailed below).

## Discussion

### Microarray performance

In our first attempt to monitor the global gene expression profile after LPS stimulation in channel catfish spleen, we have constructed a high-density oligonucleotide microarray containing almost 19,000 catfish unique sequences. In the present research, we used real-time RT-PCR to verify the microarray results. The analysis revealed a strong positive correlation between the microarray and real-time RT-PCR results. The TNFα gene was selected based on its importance in the LPS pathway and also to represent genes that did not make the final "hit" list. The TNFα microarray results failed to pass our quality control criteria, so no ratios were reported. After examination of the oligo sequences for TNFα on the microarray, we found only one of the ten probe sets demonstrated a significant difference in signal intensity between the perfectly matched and mismatched oligos. Five of the TNFα probe sets contained higher signal intensities in the mismatched than in the perfectly matched oligo. The automated oligo design software extracted all ten oligo sets from the most 5' end of the 255 bp TNFα cDNA sequence, so the discrepancy between real-time RT-PCR and microarray results for TNFα was likely due to undesirable oligo design. In fact, the one "good" oligo pair would have identified up-regulation of TNFα by LPS. This observation highlights the importance of high quality oligo design software, and that the ability to identify and eventually remove problematic oligo probes is critical to the collection of robust microarray data and minimization of discordant gene expression data from different technologies.

### Differential expression of catfish genes

The evolutionary divergence between mammalian and fish species is considerable, and we do not know if LPS can induce the IKK-NF-κB pathway and MAPK pathways (the ERK, JNK, and p38 pathways) in fish as in mammals. Although LPS-responsive cis-elements have been identified in the 5'-flanking region of many mammalian genes [20], none have been reported in catfish to date. The present research demonstrated increased expression of the p100 subunit of NF-κB at 4 h post-LPS exposure, but a sequence matching NF-κB inhibitor alpha-like proteins A and B were also significantly induced at 2 h post-LPS exposure. NF-κB inhibitor gene expression was also increased in mycobacterium-infected zebrafish [16] and bacterially infected macrophage cells from Atlantic salmon and rainbow trout [12,14]. There are instances in biological systems that a compound can up-regulate both an enzyme and its inhibitor. For example, the short-chained fatty acid butyrate up-regulated both IGF2 and IGF binding proteins IGFBP2 and IGFBP3 in bovine kidney epithelial cells (R.L., unpublished). An alternative explanation to the present observation is that the inhibitor encoded a protein with a function different from that which was annotated due to sequence similarity.

Toll-like receptor 5 demonstrated significant levels of induction at 4 h post-LPS treatment. In mammals, TLR4 is the extracellular LPS receptor whereas TLR5 recognizes flagellin [21,22]. The up-regulation of TLR5 by Gram-negative, virulent *Edwardsiella ictaluri *has been independently observed in channel catfish [[23]; Li, unpublished]. This gene was also shown to increase in the liver of Atlantic salmon exposed to the bacterium *A. salmonicida *[15], and in zebrafish exposed to the mycobacterium *M. marinum *[24]. Toll-like receptor 3 was also included on the catfish microarray, but expression was not significantly altered due to LPS exposure. The present data add evidence to the hypothesis that, in fish, microbial products may induce other TLRs than those specific for their own recognition [24].

Interferon regulatory factor 1 (IRF-1) is one of two DNA-binding transcription factors mediating Type 1 interferon gene expression. In rainbow trout gonadal cells, IRF-1 is expressed constitutively and up-regulated by poly I:C but not by LPS [25]. However, microarray experiments using Japanese flounder have demonstrated stimulation of IRF-1 expression in cultured kidney cells after exposure to LPS [11] and in kidney tissue after exposure of fish to the viral hemorrhagic septicemia virus G protein [13].

Apolipoprotein E (ApoE) has been suggested to have a regulatory role in maintaining a critical balance of various proinflammatory cytokines in mice [26] in addition to its key role in lipid metabolism. Expression of ApoE decreases in CaCo-2 cells 24 h after LPS exposure [27], and this was consistent with our observations.

The chemokine receptor CXCR4 gene selected for analysis by quantitative real-time PCR (IpCG06125) was first up-regulated by LPS at 2 h, rapidly down-regulated at 4 h, and remained down-regulated at 8 h and 24 h [see Additional file 3]. A CD14-independent model for LPS recognition has been proposed that includes a LPS activation cluster complex containing CXCR4, heat shock proteins 70 and 90, and growth differentiation factor 5 [28,29]. In addition, CXCR4 is a functional co-receptor for HIV-1 infection of human macrophages. LPS down-regulates the expression of CXCR4 on monocyte-derived macrophages [30], and the present research is consistent with this observation.

While the majority of the catfish sequences could not be annotated due to sequence identify, several catfish genes could be compared with other fish experiments utilizing microarrays to measure gene expression after experimental infection. The interleukin 1β gene demonstrated the greatest increase in LPS-induced expression in catfish spleen, and this gene was highly induced in flounder kidney cells exposed to LPS [11]. Expression of the immune responsive protein 1 gene increased in catfish spleen, and bacterially infected salmon kidneys and macrophages [14]. Catfish MHC class II gene expression decreased after LPS exposure, and this was also observed in mycobacterium-infected zebrafish [16] and salmon macrophage cells exposed to LPS [12], but LPS-treated flounder kidney cells demonstrated no change in MHC class II gene expression [11]. Immunoglobulin gene expression decreased in catfish spleen after LPS exposure and in bacterially infected Atlantic salmon macrophage cells [14], but it increased in zebrafish after mycobacterial infection [16] and in Atlantic salmon liver and kidney after bacterial infection [14,15]. Likewise, complement C3 gene expression was decreased in catfish spleen and salmon macrophages [14], but increased in kidneys from infected salmon [14] and in infected zebrafish [16]. While the present experiment demonstrated similarities and differences in species-specific gene expression after bacterial or LPS exposure, the lack of fully annotated sequences on the catfish microarray hindered a more comprehensive comparative analysis. Evaluation of immune responses across species would also enhanced by a reference set of microarray probes on each species-specific microarray.

Overall, there was no correlation between genes that were more abundant in catfish spleen (measured as number of cDNA transcripts in [31]) and genes that demonstrated significant changes in level of expression after LPS stimulation in the present research. While a 2-fold difference in expression is an arbitrary value, there are, undoubtedly, significant physiological and immunological consequences to differences in gene expression less than 2-fold. This differential reflects the inability of current microarray technology to significantly resolve smaller differences in levels of gene expression. The present research exposed difficulties inherent in performing microarray analyses from tissues *in vivo*. Differences observed between individuals within each treatment, due to asynchronized sampling and/or genetic variation, led to averaged effects which could have masked significant responses in some individuals. Nonetheless, microarrays are powerful tools that permit the simultaneous screening of thousands of genes to provide candidates for further study. While the current catfish microarray is limited by the large number of "unknown" candidate sequences, both functional experimentation and comparative genomic analyses should help improve and identification of the large number of catfish ESTs currently without annotation.

From the patterns of gene expression in this experiment, we hypothesize the observed gene expression profiles in LPS-stimulated catfish spleen result mainly from macrophage activation by LPS. However, contributions from other cell types in spleen cannot be discounted. Since macrophages play a key role in a variety of inflammatory diseases and in host defense such as pathogen phagocytosis, antigen presentation to immunocytes, and production of numerous inflammatory mediators, our future work should focus on homogeneous cell populations derived either from cultured cell lines or from macrophages isolated directly from the tissues with techniques such as laser-capture microdissection.

## Conclusion

The oligonucleotide microarray produced in the present research contained genes expressed in seventeen juvenile tissues and all neonatal tissues and provided a platform for global analysis of catfish gene expression. Levels of expression measured on the microarray were significantly correlated with levels measured by quantitative RT-PCR. Only 38% of the catfish sequences could be presently annotated, but *de novo *synthesis of oligonucleotides on the platform provides flexibility for adding annotated catfish sequences to the microarray as they become available.

The present experiment focused on the identification of differential gene expression in spleen in response to LPS injection. The innate immune response to Gram-negative bacteria is influenced by the ability of the host to respond to the LPS component, the major virulence factor of these bacteria. The present research identified 138 differentially expressed sequences that can provide clues as to the response of the catfish immune system to LPS from gram-negative bacteria, and contribute to discerning the NF-κB pathway in teleosts. Dissecting the LPS signal pathway is important in expanding our knowledge of molecular interactions between invading bacteria and host cells. Understanding the difference in the LPS pathway between fish and mammalian species will also facilitate understanding of the evolution of innate immune systems. Knowledge of the catfish immune system will help researchers identify key pathways in pathogen-induced diseases in catfish.

## Methods

### Experimental fish and LPS treatment

Channel catfish (USDA103 strain, average weight ~200 g) were maintained in a single 160-liter tank at the Catfish Genetics Research Unit. Fish were fed a commercial diet to apparent satiation daily until 24 h prior to treatment with lipopolysaccharide (LPS). Thirty-two fish were anesthetized with tricaine methanesulfonate (MS222, 100 mg/L) and randomly divided into two groups, control and LPS. There were four individuals in each of time-matched control and LPS treatment time points. Lipopolysaccharide derived from E. coli 0127:B8 (Sigma-Aldrich, St. Louis, MO) was resuspended at 1.0 mg/ml in phosphate buffered saline (PBS, pH 7.0). Fish in the LPS group each received a 0.5 ml intraperitoneal (IP) injection of LPS at a dosage of 2.5 mg/kg body weight. Fish in the control group each received a 0.5 ml IP injection of PBS. Fish were then killed by anesthesia overdose (300 mg/L) at 2, 4, 8, or 24 h post-injection and the spleen was dissected and placed in 1.0 ml Trizol (Invitrogen Corporation, Carlsbad, CA). Samples were snap-frozen in liquid nitrogen and stored at -80°C until RNA isolation. Due to expense, only two fish per treatment/time combination were used for microarray analysis, but all four fish per treatment/time combination were used for real-time RT-PCR analysis. All experimental procedures were conducted in accordance with the principles and procedures approved by the Institutional Animal Care and Use Committee, USDA-ARS Catfish Genetics Research Unit.

### Total RNA purification and generation of biotin-labelled complementary RNA

Total RNA was extracted using manufacturer's recommendations (Invitrogen), treated with 4–10 units DNase I (Ambion Inc., Austin, TX) per 100 μg total RNA at 37°C for 30 min, then further purified using an RNeasy Mini kit (Qiagen Inc., Valencia, CA). Equal masses of total RNA were pooled from the eight individuals in the control group (two fish in each of four time-matched controls). This pool of RNA was labelled as described below and served as a control in the microarray hybridization. The total RNA from the eight LPS-stimulated fish (two in each of four time points) was not pooled.

Biotin-labelled complementary RNA (cRNA) was generated using a modified procedure for the Superscript Choice System (Invitrogen) for double-stranded (ds) cDNA synthesis followed by *in vitro *transcription. First strand cDNA was synthesized by SuperScript II reverse transcriptase from 2.5 μg of total RNA using 100 pmoles of T7 promoter Oligo dT primer. After ds cDNA synthesis, the cDNA was purified with DNA Clean & Concentrator-5 (Zymo Research, Orange, CA), eluted with 8–16 μl of ddH_2_O, and concentrated to 3 μl by vacuum centrifugation. Complementary RNA was then synthesized with a MEGAscript *in vitro *Transcription kit (Ambion). The 23.0 μl *in vitro *transcription reaction included the entire 3.0 μl of ds cDNA, 2.3 μl of 10× Ambion reaction buffer, 2.3 μl of 10× Ambion T7 enzyme mix, and 15.4 μl of NTP labelling mix (7.5 mM ATP, 7.5 mM GTP, 5.625 mM UTP, 5.625 mM CTP and 1.875 mM biotin-16-UTP and 1.875 mM biotin-11 CTP). The *in vitro *transcription reaction was incubated at 37°C for 16 hours in a thermal cycler. Transcribed cRNA was then purified with an RNeasy Mini kit. Generally, 40–60 μg of cRNA was obtained from 2.5 μg original RNA. The sample integrity of the cRNA was verified with an Agilent 2100 Bioanalyzer (Agilent Technologies, Inc., Palo Alto, CA). The biotinylated cRNA products were then fragmented to 50–200 bp prior to hybridization by heating cRNA in 1× fragmentation buffer (40 mM Tris-acetate, pH.8.1, 100 mM potassium acetate, 30 mM magnesium acetate) at 95°C for 35 min.

### Catfish oligonucleotide microarray production, hybridization, and image acquisition

Published sequences from cDNA libraries were obtained from the TIGR catfish gene index release 5.0 [19]. These ESTs were derived from brain, gill, intestine, liver, kidney (anterior and posterior), macrophage, muscle, olfactory tissue, ovary, peripheral blood leukocytes, pituitary, skin, spleen, stomach, taste tissue, and testis. An additional 9,821 novel sequences were obtained by single-pass directional sequencing of normalized cDNA libraries produced from mRNA obtained from 5 d, 9 d, and 14 d whole fry. The fry and TIGR gene index sequences were clustered using CAP3 software [32] and the resulting contigs and singlets were assigned an IpCG designation (*Ictalurus punctatus *Catfish Genetics). Subsequent to the production of the microarray, the sequence data from the fry cDNA libraries were submitted to GenBank as accessions CV987367 – CV997187 and were incorporated into release 6.0 of the TIGR gene index. The tables were updated to include the release 6.0 Tentative Consensus sequence (TC) designation of these sequences where applicable. The catfish ESTs were compared against the GenBank non-redundant peptide database using BlastX, and only those with E value = 0.005 (minimum bit score = 100) were annotated.

Microarray production, hybridization, and imaging were performed by NimbleGen Systems Inc. (Madison, WI). A total of 18,989 unique catfish ESTs were used to construct the high-density catfish oligonucleotide DNA microarrays. The microarrays were synthesized *in situ *using photo deprotection chemistry with the Maskless Array Synthesizer system [33]. Twenty 24-mer oligonucleotides (features) were designed to represent each EST; ten were perfectly matched oligos positioned throughout the sequence, and these ten were duplicated but with two mismatched bases at positions #13 and #19. The feature size for the microarray was 16 μM × 16 μM, and there were 382,409 features within the 17 mm × 13 mm array area. Of these, 379,652 were catfish-specific, and the remaining 2,757 were used for quality control of oligonucleotide synthesis and hybridization, and for signal normalization.

The microarrays were pre-hybridized with 1X MES hybridization buffer (100 mM MES, 1.0 M Na+, 20 mM EDTA, 0.01% Tween20) plus 40 μg of herring sperm DNA and 200 μg of acetylated BSA at 45°C for 15 min, followed by hybridization in the same buffer with 10 μg of denatured and fragmented cRNA per microarray at 45°C for 16–20 h with constant rotation. After hybridization, the microarrays were immediately washed extensively with non-stringent wash buffer (6X SSPE, 0.01% Tween20) at room temperature, then with stringent wash buffer (100 mM MES salt and free acid solution, 0.1 M Na+, 0.01% Tween20) at 45°C. After final rinsing with non-stringent wash buffer, the microarrays were stained with 1X Stain buffer (100 mM MES, 1 M Na+, 0.05% Tween20, 50 mg/ml of BSA, and 1 mg/ml of Cy3-streptavidin) at room temperature for 25 min. After the stain buffer was removed, the microarrays were rinsed with non-stringent wash buffer and immediately dried under argon gas. The microarrays were scanned with an Axon GenePix 4000B scanner (Molecular Devices Corp., Union City, CA) at 5 μM resolution. The data were extracted from the raw images with NimbleScan software (Nimblegen, Inc.). A total of ten microarrays were used for this project: two replicates for the control pool and one microarray for each of the eight LPS-stimulated individuals.

### Data analysis, statistics, and bioinformatics

Relative signal intensity (log2 transformed) for each feature was generated using the Robust Multi-Array Average (RMA) algorithm [34]. The signal intensity was background corrected based on the quantile normalization process [35,36] for all microarrays from the entire experiment (under uniform conditions) using data from only perfectly matched oligos. Only oligos with a ratio of signal to global average background greater than 2.0, with a perfectly matched signal 150% of the mismatched signal, and with a correlation > 0.9 between biological replicates were included. The normalized data was analyzed using Significance Analysis of Microarrays [37] from the TIGR Multiexperiment Viewer analysis software package [38] with two-class unpaired design for each time point. The RMA value from perfectly matched oligos was used to calculate fold differences as the average signal intensity of treated animals divided by the average signal intensity of control animals. The list of significant genes that changed at least 2-fold was generated with an estimated global false discovery rate <10%. Differentially expressed sequences were compared to the Ensembl peptide databases derived from the zebrafish (v.5) and Tetraodon (v.7) genomes [39] using BlastX (E-value cutoff 0.005, minimum bit score = 100) and annotated accordingly [see Additional file 1 and Additional file 2].

### Real-time RT-PCR

Real-time RT-PCR analysis was carried out with the TaqMan One-Step RT-PCR kit (Applied Biosystems, Foster City, CA) in a 25 μL reaction volume containing 200 nM each amplification primer, 100 nM dual-labelled probe [see Additional file 2], and 200 ng of total RNA. Products were amplified in an iCycler iQ™ Real Time PCR Detection System (Bio-Rad Laboratories, Hercules, CA) with the following profile: 48°C for 30 min; 95°C for 10 min; 40 cycles of 95°C for 30s, 60°C for 1 min. Expression levels of β-actin were used as endogenous controls within each sample [see Additional file 2]. Relative levels of gene expression were calculated using the 2^ΔΔCT^ method [40].

## Authors' contributions

RWL conceived the study, carried out the experiments, provided initial data analysis, and drafted the manuscript. GCW designed the microarray, participated in data analysis, and co-wrote the manuscript. All authors read and approved the final manuscript.

## Supplementary Material

Additional File 1 Genes up-regulated at least 2-fold after LPS exposure. Click here for file

Additional File 2 Genes down-regulated at least 2-fold after LPS exposure. Click here for file

Additional File 3 Comparison of relative gene expression levels between microarray and real-time RT-PCR platforms. Click here for file

## References

[B1] Miya M, Nishida M (2000). Use of mitogenomic information in teleostean molecular phylogenetics: a tree-based exploration under the maximum-parsimony optimality criterion. Molec Phylogenet Evol.

[B2] Elmerot C, Arnason U, Gojobori T, Janke A (2002). The mitochondrial genome of the pufferfish, *Fugu rubripes*, and ordinal teleostean relationships. Gene.

[B3] USDA Catfish Production Report.

[B4] Shen L, Stuge TB, Zhou H, Khayat M, Barker KS, Quiniou SMA, Wilson M, Bengtén E, Chinchar VG, Clem LW, Miller NW (2002). Channel catfish cytotoxic cells: a mini-review. Dev Comp Immunol.

[B5] Ton C, Stamatiou D, Dzau VJ, Liew CC (2002). Construction of a zebrafish cDNA microarray: gene expression profiling of the zebrafish during development. Biochem Biophys Res Commun.

[B6] Mathavan S, Lee SG, Mak A, Miller LD, Murthy KR, Govindarajan KR, Tong Y, Wu YL, Lam SH, Yang H, Ruan Y, Korzh V, Gong Z, Liu ET, Lufkin T (2005). Transcriptome analysis of zebrafish embryogenesis using microarrays. PLoS Genet.

[B7] Gracey AY, Troll JV, Somero GN (2001). Hypoxia-induced gene expression profiling in the euryoxic fish *Gillichthys mirabilis*. Proc Natl Acad Sci USA.

[B8] Ton C, Stamatiou D, Liew CC (2003). Gene expression profile of zebrafish exposed to hypoxia during development. Physiol Genomics.

[B9] Gracey AY, Fraser EJ, Li W, Fang Y, Taylor RR, Rogers J, Brass A, Cossins AR (2004). Coping with cold: An integrative, multitissue analysis of the transcriptome of a poikilothermic vertebrate. Proc Natl Acad Sci USA.

[B10] Krasnov A, Koskinen H, Pehkonen P, Rexroad CE, Afanasyev S, Molsa H (2005). Gene expression in the brain and kidney of rainbow trout in response to handling stress. BMC Genomics.

[B11] Kurobe T, Yasuike M, Kimura T, Hirono I, Aoki T (2005). Expression profiling of immune-related genes from Japanese flounder *Paralichthys olivaceus *kidney cells using cDNA microarrays. Dev Comp Immunol.

[B12] Mackenzie S, Iliev D, Liarte C, Koskinen H, Planas JV, Goetz FW, Molsa H, Krasnov A, Tort L (2005). Transcriptional analysis of LPS-stimulated activation of trout (*Oncorhynchus mykiss*) monocyte/macrophage cells in primary culture treated with cortisol. Mol Immunol.

[B13] Byon JY, Ohira T, Hirono I, Aoki T (2005). Use of a cDNA microarray to study immunity against viral hemorrhagic septicemia (VHS) in Japanese flounder (*Paralichthys olivaceus*) following DNA vaccination. Fish Shellfish Immunol.

[B14] Rise ML, Jones SR, Brown GD, von Schalburg KR, Davidson WS, Koop BF (2004). Microarray analyses identify molecular biomarkers of Atlantic salmon macrophage and hematopoietic kidney response to *Piscirickettsia salmonis *infection. Physiol Genomics.

[B15] Ewart KV, Belanger JC, Williams J, Karakach T, Penny S, Tsoi SC, Richards RC, Douglas SE (2005). Identification of genes differentially expressed in Atlantic salmon (*Salmo salar*) in response to infection by Aeromonas salmonicida using cDNA microarray technology. Dev Comp Immunol.

[B16] Meijer AH, Verbeek FJ, Salas-Vidal E, Corredor-Adamez M, Bussman J, van der Sar AM, Otto GW, Geisler R, Spaink HP (2005). Transcriptome profiling of adult zebrafish at the late stage of chronic tuberculosis due to *Mycobacterium marinum *infection. Mol Immunol.

[B17] Liu Z (2003). A review of catfish genomics: progress and perspectives. Comp Funct Genom.

[B18] Nonneman D, Waldbieser GC (2005). Isolation and enrichment of abundant microsatellites from a channel catfish (*Ictalurus punctatus*) brain cDNA library. Anim Biotechnol.

[B19] The Institute for Genomic Research. http://www.tigr.org/tigr-scripts/tgi/T_index.cgi?species=catfish.

[B20] Sweet MJ, Hume DA (1996). Endotoxin signal transduction in macrophages. J Leukoc Biol.

[B21] Poltorak A, He X, Smirnova I, Liu MY, Van Huffel C, Du X, Birdwell D, Alejos E, Silva M, Galanos C, Freudenberg M, Ricciardi-Castagnoli P, Layton B, Beutler B (1998). Defective LPS signaling in C3H/HeJ and C57BL/10ScCr mice: mutations in Tlr4 gene. Science.

[B22] Takeuchi O, Hoshino K, Kawai T, Sanjo H, Takada H, Ogawa T, Takeda K, Akira S (1999). Differential roles of TLR2 and TLR4 in recognition of gram-negative and gram-positive bacterial cell wall components. Immunity.

[B23] Bilodeau AL, Waldbieser GC (2005). Activation of TLR3 and TLR5 in channel catfish exposed to virulent *Edwardsiella ictaluri*. Dev Comp Immunol.

[B24] Meijer AH, Krens SF, Medina Rodriguez IA, He S, Bitter W, Snaar-Jagalska BE, Spaink HP (2004). Expression analysis of the Toll-like receptor and TIR domain adaptor families of zebrafish. Mol Immunol.

[B25] Collet B, Secombes CJ (2002). Type I-interferon signalling in fish. Fish Shellfish Immunol.

[B26] Harry GJ, Lefebvre d'Hellencourt C, Bruccoleri A, Schmechel D (2000). Age-dependent cytokine responses: trimethyltin hippocampal injury in wild-type, APOE knockout, and APOE4 mice. Brain Behav Immun.

[B27] Ripolles Piquer B, Nazih H, Neunlist M, Huvelin JM, Bard JM (2004). Effect of LPS on basal and induced apo E secretion by 25-OH chol and 9cRA in differentiated CaCo-2. J Cell Biochem.

[B28] Triantafilou K, Triantafilou M, Dedrick RL (2001). A CD14-independent LPS receptor cluster. Nat Immunol.

[B29] Triantafilou M, Triantafilou K (2002). Lipopolysaccharide recognition: CD14, TLRs and the LPS-activation cluster. Trends Immunol.

[B30] Verani A, Sironi F, Siccardi AG, Lusso P, Vercelli D (2002). Inhibition of CXCR4-tropic HIV-1 infection by lipopolysaccharide: evidence of different mechanisms in macrophages and T lymphocytes. J Immunol.

[B31] Kocabas AM, Li P, Cao D, Karsi A, He C, Patterson A, Ju Z, Dunham RA, Liu Z (2002). Expression profile of the channel catfish spleen: Analysis of genes involved in immune functions. Mar Biotechnol.

[B32] Huang X, Madan A (1999). CAP3: A DNA sequence assembly program. Genome Res.

[B33] Singh-Gasson S, Green RD, Yue Y, Nelson C, Blattner F, Sussman MR, Cerrina F (1999). Maskless fabrication of light-directed oligonucleotide microarrays using a digital micromirror array. Nat Biotechnol.

[B34] Irizarry RA, Hobbs B, Collin F, Beazer-Barclay YD, Antonellis KJ, Scherf U, Speed TP (2003). Exploration, normalization, and summaries of high density oligonucleotide array probe level data. Biostatistics.

[B35] Bolstad B, Irizarry R, Astrand M, Speed T (2003). A comparison of normalization methods for high density oligonucleotide array data based on variance and bias. Bioinformatics.

[B36] Bioconductor Open Source Software for Bioinformatics. http://www.bioconductor.org.

[B37] Tusher VG, Tibshirani R, Chu G (2001). Significance analysis of microarrays applied to the ionizing radiation response. Proc Natl Acad Sci USA.

[B38] TIGR Microarray software. http://www.tigr.org/software/microarray.shtml.

[B39] Ensembl Genome Browser Zebrafish (*Danio rerio*) assembly version 5 and Spotted Green Pufferfish (*Tetraodon nigroviridis*) assembly version 7. http://www.ensembl.org/index.html.

[B40] Livak KJ, Schmittgen TD (2001). Analysis of relative gene expression data using Real-Time quantitative PCR and the 2^ΔΔ*CT *^method. Methods.

