# Supplementary material for: Production and utilization of a high-density oligonucleotide microarray in channel catfish, Ictalurus punctatus
Source: BMC Genomics. 2006 Jun 1;7:134. doi: 10.1186/1471-2164-7-134 (PMC1501020; doi:10.1186/1471-2164-7-134)
Supplement: Additional File 1 — Genes up-regulated at least 2-fold after LPS exposure. [file 1471-2164-7-134-S1.doc]

**Table 1. Genes up-regulated at least 2-fold after LPS exposure.**

| **Locus** | **Accession** | | **Gene** | **Time** | **q-**  **Value** | **Ensembl match** | **eValue** |
| --- | --- | --- | --- | --- | --- | --- | --- |
| IpCG03626 | CV989141 | | CD83 antigen | 2h | 0.00 | ENSDARP-  00000042302 | 7.9e-34 |
| IpCG03141 | CV987587 | | NF-κB inhibitor alpha-like protein B | 2h | 4.55 | ENSDARP-  00000015303 | 2.0e-53 |
| IpCG06517 | TC7802 | | Interleukin-1ß | 2h, 4h | 0.00 | ENSDARP-  00000002293 | 5.4e-42 |
| IpCG06765 | TC8130 | | CCL4 | 2h, 4h | 4.55 | ENSDARP-  00000067214 | 3.3e-15 |
| IpCG05035 | CV993724 | | Toll-like receptor5 | 2h, 4h | 0.00 | GSTENP-  00011330001 | 1.9e-44 |
| IpCG02008 | CV990051 | | NF-κB inhibitor alpha-like protein A | 2h, 4h | 0.00 | ENSDARP-  00000007861 | 1.0e-82 |
| IpCG08697 | BM425354 | | Novel protein similar to vertebrate basic leucine zipper transcription factor, ATF-like | 4h | 0.00 | ENSDARP-  00000014497 | 5.5e-08 |
| IpCG01375 | TC9004 | | Small chemokine, CXC/Interleukin 8 like | 4h | 0.00 | ENSDARP-  00000051136 | 7.0e-22 |
| IpCG05333 | CV994911 | | G-protein coupled receptor | 4h | 3.53 | ENSDARP-  00000060553 | 1.4e-32 |
| IpCG01664 | TC8889 | | Glutamate receptor ionotropic N-methyl D-asparate-associated protein 1 | 4h | 1.21 | ENSDARP-  00000028496 | 9.2e-29 |
| IpCG02613 | CB938981 | | A disintegrin and metalloproteinase domain 8 | 4h | 0.00 | ENSDARP-  00000001187 | 2.5e-19 |
| IpCG00278 | CV989308 | | Interferon regulatory factor 1 | 4h | 0.00 | ENSDARP-  00000048292 | 6.7e-56 |
| IpCG06957 | TC8567 | | Leukocyte cell-derived chemotaxin 2 precursor (Chondromodulin II) | 4h | 5.24 | ENSDARP-  00000038063 | 5.5e-54 |
| IpCG06941 | TC8532 | | Neutrophil cytosolic factor 1 | 4h | 0.00 | ENSDARP-  00000049873 | 1.3e-139 |
| IpCG16438 | CB939408 | | Nuclear factor NF-κB p100 subunit | 4h | 0.00 | ENSDARP-  00000057671 | 1.9e-64 |
| IpCG15485 | BM438717 | | TNFα-induced adipose-related protein | 4h | 3.45 | ENSDARP-  00000059039 | 1.0e-13 |
| **Table 1, cont’d** | |  |  |  |  |  |  |
| **Locus** | **Accession** | | **Gene** | **Time** | **q**  **Value** | **Ensembl match** | **eValue** |
| IpCG05900 | TC6684 | | Transcription factor AP-1 (Proto-oncogene c-jun) | 4h | 0.00 | ENSDARP-  00000009150 | 1.9e-16 |
| IpCG05989 | TC6983 | | Macrophage myristoylated alanine-rich C kinase substrate | 4h | 0.00 | ENSDARP-  00000051803 | 8.3e-20 |
| IpCG09242 | BM495436 | | Hypothetical protein LOC503741 protein (Immunoglobulin like) | 4h | 1.24 | ENSDARP-  00000061544 | 7.8e-09 |
| IpCG05825 | CV997012 | | Immune responsive protein 1 | 4h | 0.00 | ENSDARP-  00000035264 | 2.5e-20 |
| IpCG10289 | CF263575 | | Vacuole membrane protein 1 | 4h | 4.38 | GSTENP-  00005027001 | 2.1e-12 |
| IpCG07829 | TC9831 | | VHSV-induced protein-6 | 4h | 5.79 | ENSDARP-  00000066996 | 9.5e-68 |
| IpCG11302 | CK413274 | | Zinc finger, A20 Domain containing 1 protein | 4h | 7.36 | ENSDARP-  00000041810 | 2.0e-05 |
| IpCG10849 | CK412191 | | Ornithine decarboxylase 1 | 4h | 7.62 | ENSDART-  00000017510 | 2.4e-06 |
| IpCG07975 | BE212968 | | Gap junction alpha-1 protein | 4h | 1.27 | ENSDART-  00000061261 | 4.4e-19 |
| IpCG04728 | CV992598 | | Myristoylated alanine-rich C kinase substrate | 4h | 0.00 | ENSDART-  00000051804 | 4.3e-109 |
| IpCG15378 | BM029583 | | No homology | 2h | 0.00 |  |  |
| IpCG19854 | CK420469 | | No homology | 2h | 4.55 |  |  |
| IpCG20673 | CK423912 | | No homology | 2h | 5.36 |  |  |
| IpCG20772 | CK424226 | | No homology | 2h | 0.00 |  |  |
| IpCG13253 | CK420039 | | No homology | 2h, 4h | 5.56 |  |  |
| IpCG03158 | CV987628 | | No homology | 4h | 0.00 |  |  |
| IpCG03238 | CV987846 | | No homology | 4h | 5.91 |  |  |
| IpCG03632 | CV989153 | | No homology | 4h | 5.02 |  |  |
| IpCG04226 | CV990867 | | No homology | 4h | 7.02 |  |  |
| IpCG04481 | CV991696 | | No homology | 4h | 0.00 |  |  |
| IpCG04689 | CV992451 | | No homology | 4h | 7.36 |  |  |
| IpCG07575 | TC9503 | | No homology | 4h | 6.37 |  |  |
| **Table 1, cont’d** | |  |  |  |  |  |  |
| **Locus** | **Accession** | | **Gene** | **Time** | **q**  **Value** | **Ensembl match** | **eValue** |
| IpCG08242 | BE469766 | | No homology | 4h | 0.00 |  |  |
| IpCG08286 | BE470169 | | No homology | 4h | 0.00 |  |  |
| IpCG08469 | BM424525 | | No homology | 4h | 0.00 |  |  |
| IpCG08531 | BM424770 | | No homology | 4h | 0.00 |  |  |
| IpCG08690 | BM425324 | | No homology | 4h | 0.00 |  |  |
| IpCG08696 | BM425350 | | No homology | 4h | 5.02 |  |  |
| IpCG08699 | BM425362 | | No homology | 4h | 4.38 |  |  |
| IpCG08850 | BM439121 | | No homology | 4h | 0.00 |  |  |
| IpCG08877 | BM439182 | | No homology | 4h | 0.00 |  |  |
| IpCG09595 | CB938302 | | No homology | 4h | 5.24 |  |  |
| IpCG09836 | CB940389 | | No homology | 4h | 0.00 |  |  |
| IpCG10280 | CF263544 | | No homology | 4h | 7.62 |  |  |
| IpCG10836 | CK412140 | | No homology | 4h | 0.00 |  |  |
| IpCG13253 | CK420039 | | No homology | 4h | 0.00 |  |  |
| IpCG14007 | CK423001 | | No homology | 4h | 1.34 |  |  |
| IpCG14871 | BE212735 | | No homology | 4h | 0.00 |  |  |
| IpCG15536 | BM438848 | | No homology | 4h | 7.15 |  |  |
| IpCG15586 | BM438945 | | No homology | 4h | 0.00 |  |  |
| IpCG15608 | BM438995 | | No homology | 4h | 0.00 |  |  |
| IpCG15623 | BM439030 | | No homology | 4h | 6.37 |  |  |
| IpCG16063 | BM496636 | | No homology | 4h | 0.00 |  |  |
| IpCG16138 | CB937040 | | No homology | 4h | 0.00 |  |  |
| IpCG17503 | CK411851 | | No homology | 4h | 6.37 |  |  |
| IpCG20324 | CK422378 | | No homology | 4h | 0.00 |  |  |
| IpCG20434 | CK423026 | | No homology | 4h | 0.00 |  |  |
| IpCG15164 | BE470366 | | No homology | 8h | 0.00 |  |  |
|  |  | |  |  |  |  |  |

Accession: Entries with TC prefix are Tentative Clusters from TIGR Catfish Gene Index, all other entries are from GenBank dbEST.

Time = Time after LPS exposure

q-Value = probability that gene identified is significant by chance.
