# Supplementary material for: Production and utilization of a high-density oligonucleotide microarray in channel catfish, Ictalurus punctatus
Source: BMC Genomics. 2006 Jun 1;7:134. doi: 10.1186/1471-2164-7-134 (PMC1501020; doi:10.1186/1471-2164-7-134)
Supplement: Additional File 2 — Genes down-regulated at least 2-fold after LPS exposure. [file 1471-2164-7-134-S2.doc]

**Table 2. Genes down-regulated at least 2-fold after LPS exposure.**

| **Locus** | **Accession** | | **Gene** | **Time** | **q-**  **Value** | **Ensembl match** | **eValue** |
| --- | --- | --- | --- | --- | --- | --- | --- |
| IpCG00386 | CV997126 | | Complement C3 | 2h | 4.29 | ENSDARP-00000052682 | 9.1e-38 |
| IpCG05545 | CV995902 | | Heat shock cognate 71 kDa protein (Heat  shock 70 kDa protein 8) | 2h | 4.29 | ENSDARP-00000042383 | 2.9e-56 |
| IpCG05759 | CV996728 | | MID1 interacting protein 1 | 2h | 5.56 | ENSDARP-00000051006 | 3.4e-10 |
| IpCG01812 | NP857000 | | Immunoglobulin heavy chain variable region | 2h | 4.29 | ENSDARP-00000055249 | 1.7e-34 |
| IpCG02688 | AAA16660 | | Immunoglobulin light chain | 2h | 5.77 | ENSDARP-00000049837 | 5.7e-49 |
| IpCG02108 | AAA82594 | | Immunoglobulin light chain F class | 2h | 8.54 | ENSDARP-00000049837 | 1.6e-64 |
| IpCG15664 | BM494359 | | Lipoprotein lipase | 2h | 7.50 | ENSDARP-00000062549 | 5.0e-13 |
| IpCG04499 | CV991749 | | Predicted: hypothetical protein XP_679163 | 2h | 0.00 | XP_684255 | 4.0e-11 |
| IpCG04908 | CV993251 | | Diacylglycerol acyltransferase | 2h | 4.29 | ENSDARP-00000042516 | 1.1e-27 |
| IpCG15427 | BM424290 | | SRY (sex determining region Y)-box 4 | 2h | 2.63 | ENSDART-00000056660 | 1.7e-61 |
| IpCG20631 | CK423382 | | Secreted acidic cysteine rich glycoprotein | 2h | 5.26 | ENSDART-00000039660 | 7.5e-25 |
| IpCG01225 | TC6870 | | Apolipoprotein Eb | 2h, 8h | 0.00 | ENSDARP-00000058964 | 2.5e-88 |
| IpCG04567 | CV991959 | | Tissue inhibitor of metalloproteinase 4 | 2h, 24h | 5.77 | ENSDARP-00000066231 | 2.8e-27 |
| IpCG08302 | BM027940 | | Adipose differentiation-related protein | 8h | 6.67 | ENSDARP-00000061955 | 6.8e-08 |
| IpCG13898 | CK422794 | | Leiomodin 1 | 8h | 6.67 | ENSDARP-00000067692 | 6.6e-22 |
| IpCG00885 | CV994441 | | Complement C3-S | 8h | 6.67 | ENSDARP-00000052682 | 6.8e-13 |
|  |  | |  |  |  |  |  |
| **Table 2, cont’d** | |  |  |  |  |  |  |
| **Locus** | **Accession** | | **Gene** | **Time** | **q**  **Value** | **Ensembl match** | **eValue** |
| IpCG06864 | TC8263 | | NK lysin-like protein | 8h | 0.00 | XP_689995 | 2.0e-16 |
| IpCG07002 | TC8671 | | Hemoglobin alpha adult-1 | 8h | 4.17 | ENSDARP-00000053077 | 2.1e-36 |
| IpCG03493 | CV988725 | | C-ets proto-oncogene | 8h | 7.81 | ENSDARP-00000004618 | 1.4e-64 |
| IpCG20768 | CK424211 | | Nuclear 1 P8 Candidate of Metastasis 1 | 8h | 5.56 | ENSDARP-00000039464 | 4.3e-22 |
| IpCG07512 | TC9415 | | CXC chemokine receptor | 8h, 24h | 0.00 | GSTENP-00004498001 | 8.6e-25 |
| IpCG19896 | CK420555 | | 3'5'-cyclic nucleotide phosphodiesterase | 8h, 24h | 3.33 | GSTENP-00030267001 | 5.4e-3 |
| IpCG06060 | TC7140 | | 5'-nucleotidase, cytosolic II | 24h | 6.17 | ENSDARP-00000057573 | 8.7e-132 |
| IpCG13734 | CK422021 | | Interleukin-8 | 24h | 0.00 | ENSDARP-00000051135 | 3.0e-06 |
| IpCG01650 | TC8797 | | MHC class II alpha chain | 24h | 7.87 | ENSDARP-00000010619 | 4.3e-54 |
| IpCG01162 | TC8969 | | MHC class II alpha chain | 24h | 0.00 | ENSDARP-00000018164 | 4.2e-49 |
| IpCG17444 | CK411143 | | MHC class II antigen | 24h | 0.00 | ENSDARP-00000043524 | 4.7e-09 |
| IpCG05341 | CV994946 | | Acid phosphatase 5, tartrate resistant | 24h | 0.00 | ENSDARP-00000017532 | 6.3e-28 |
| IpCG20605 | CK423335 | | Invariant chain-like protein 2 | 24h | 8.71 | ENSDARP-00000053204 | 7.2e-15 |
| IpCG09462 | CB937692 | | No homology | 2h | 5.77 |  |  |
| IpCG14891 | BE212788 | | No homology | 2h | 2.63 |  |  |
| IpCG15131 | BE470304 | | No homology | 2h | 4.29 |  |  |
| IpCG15835 | BM494752 | | No homology | 2h | 5.26 |  |  |
| IpCG17175 | CK410098 | | No homology | 2h | 7.50 |  |  |
|  |  | |  |  |  |  |  |
| **Table 2, cont’d** | |  |  |  |  |  |  |
| **Locus** | **Accession** | | **Gene** | **Time** | **q**  **Value** | **Ensembl match** | **eValue** |
| IpCG18785 | CK416478 | | No homology | 2h | 5.77 |  |  |
| IpCG20164 | CK421565 | | No homology | 2h | 0.00 |  |  |
| IpCG20015 | CK421238 | | No homology | 2h | 0.00 |  |  |
| IpCG03618 | CV989124 | | No homology | 2h, 4h, 8h | 0.00 |  |  |
| IpCG05047 | CV993780 | | No homology | 2h, 4h, 8h | 0.00 |  |  |
| IpCG05808 | CV996924 | | No homology | 2h, 8h | 4.29 |  |  |
| IpCG08016 | BE213060 | | No homology | 2h, 8h | 3.33 |  |  |
| IpCG09284 | BM496721 | | No homology | 2h, 8h | 2.63 |  |  |
| IpCG04059 | CV990353 | | No homology | 2h, 24h | 4.29 |  |  |
| IpCG04661 | CV992315 | | No homology | 2h, 24h | 0.00 |  |  |
| IpCG12907 | CK418316 | | No homology | 2h, 24h | 3.57 |  |  |
| IpCG16279 | CB938634 | | No homology | 2h, 24h | 2.63 |  |  |
| IpCG01816 | BE212925 | | No homology | 8h | 0.00 |  |  |
| IpCG02213 | CV989425 | | No homology | 8h | 3.33 |  |  |
| IpCG03370 | CV988281 | | No homology | 8h | 4.17 |  |  |
| IpCG04216 | CV990834 | | No homology | 8h | 0.00 |  |  |
| IpCG04530 | CV991846 | | No homology | 8h | 3.33 |  |  |
| IpCG04578 | CV992008 | | No homology | 8h | 7.69 |  |  |
| IpCG09832 | CB940351 | | No homology | 8h | 5.00 |  |  |
| IpCG10318 | CF263668 | | No homology | 8h | 4.17 |  |  |
| IpCG15006 | BE468911 | | No homology | 8h | 6.67 |  |  |
| IpCG15281 | BM029364 | | No homology | 8h | 5.26 |  |  |
| IpCG15341 | BM029519 | | No homology | 8h | 4.17 |  |  |
| IpCG15428 | BM424296 | | No homology | 8h | 6.45 |  |  |
| IpCG20512 | CK423164 | | No homology | 8h | 2.94 |  |  |
| IpCG11868 | CK414909 | | No homology | 8h, 24h | 2.94 |  |  |
|  |  | |  |  |  |  |  |

| **Table 2, cont’d** | |  |  |  |  |  |  |
| --- | --- | --- | --- | --- | --- | --- | --- |
| **Locus** | **Accession** | | **Gene** | **Time** | **q**  **Value** | **Ensembl match** | **eValue** |
| IpCG13727 | CK422011 | | No homology | 8h, 24h | 0.00 |  |  |
| IpCG20517 | CK423176 | | No homology | 8h, 24h | 0.00 |  |  |
| IpCG01508 | TC8367 | | No homology | 24h | 0.00 |  |  |
| IpCG03362 | CV988256 | | No homology | 24h | 7.81 |  |  |
| IpCG03672 | CV989264 | | No homology | 24h | 2.52 |  |  |
| IpCG09556 | CB938144 | | No homology | 24h | 0.00 |  |  |
| IpCG10664 | CK411260 | | No homology | 24h | 0.00 |  |  |
| IpCG10708 | CK411389 | | No homology | 24h | 9.43 |  |  |
| IpCG11462 | CK413515 | | No homology | 24h | 8.71 |  |  |
| IpCG13932 | CK422868 | | No homology | 24h | 2.16 |  |  |
| IpCG14022 | CK423405 | | No homology | 24h | 2.16 |  |  |
| IpCG14536 | CK425645 | | No homology | 24h | 7.81 |  |  |
| IpCG15409 | BM029650 | | No homology | 24h | 0.00 |  |  |
| IpCG17243 | CK410284 | | No homology | 24h | 0.00 |  |  |
|  |  | |  |  |  |  |  |

Accession: Entries with TC prefix are Tentative Clusters from TIGR Catfish Gene Index, all other entries are from GenBank dbEST.

Time = Time after LPS exposure

q-Value = probability that gene identified is significant by chance.
