# Supplementary material for: Production and utilization of a high-density oligonucleotide microarray in channel catfish, Ictalurus punctatus
Source: BMC Genomics. 2006 Jun 1;7:134. doi: 10.1186/1471-2164-7-134 (PMC1501020; doi:10.1186/1471-2164-7-134)
Supplement: Additional File 3 — Comparison of relative gene expression levels between microarray and real-time RT-PCR platforms. [file 1471-2164-7-134-S3.doc]

**Table 3. Comparison of relative gene expression levels between microarray and real-time RT-PCR platforms.**

Expression in the control group was arbitrarily set at 1.0. The values below 1.0 indicated down-regulation by LPS.

Genes and Primer sequences Platforms Fold Changes

Time post-LPS treatments

2 h 4 h 8 h 24 h

IpCG06765 (Macrophage inflammatory protein-1α)

PrimerF: CTGTCCATCAGTCCTTTGGGTTAG Microarray 5.4 16.7 4.0 4.2

PrimerR: AAACGCATCCCTTCAGTGAATGT Real-time PCR 6.5 33.1 3.9 3.4

Probe: CTCAGCAGCACCACCACGGCGGA

IpCG03141 (Zgc:56666 protein)

PrimerF: AACAGAACTACCAGAGACAGACG Microarray 1.8 2.9 0.9 0.7

PrimerR: CTACTAACATGGGGTCGCAGC Real-time PCR 3.5 5.9 1.4 0.9

Probe: CAGACGCTCCACCACCAGAGGCTG

IpCG06517 (IL1-ß)

PrimerF: TCAAAGGAGGACTATAAGCCAGTG Microarray 8.9 51.4 1.2 1.3

PrimerR: AGTCTCATCATGGAGTGTGAACAG Real-time PCR 9.8 84.4 0.8 0.6

Probe: ATGTGTAAGCAGCAATCCAGTCACCTCCA

IpCG07620 (TNFα)

PrimerF: TCGTGGTCTTCTTCAGGAGTTTG Microarray -- -- -- --

PrimerR: TGAGAAAAGCAGACAGCGGC Real-time PCR 7.7 22.6 1.7 1.9

Probe: AACCTGTGGAGTCCTTCTCGCCGTGG

IpCG06125 (CXCR4)

PrimerF: ACCCTGAACCTGTATAGC Microarray 2.4 0.4 0.3 0.4

PrimerR: TTAGCGAACACCAGATCA Real-time PCR 5.3 0.4 0.1 0.2

Probe: CGTCCTCATCCTCGCCTTCAT

IpCG17616 (unknown)

PrimerF: GAGCTGGCAGTCGTGGATTTAA Microarray 1.8 1.9 2.0 1.4

PrimerR: TCATTAAGCACATCGTTCAGAGTCT Real-time PCR 1.4 3.1 3.1 1.3

Probe: CCAACAGCCTCCAACAAGACCTCCGT

IpCG09809 (HTGN29)

PrimerF: CAACAGCATCCATAACACAAGCAA Microarray 1.6 1.4 1.2 1.2

PrimerR: CAGGAGAGGTGGTGGACTCAA Real-time PCR 1.0 1.6 1.3 0.9

Probe: CCCCAAACCAACGAGTGGAGCTGAGA

IpCG06019 (unknown)

PrimerF: AGATAGTAGCCTGCGATGGAAAA Microarray 0.7 1.0 0.8 0.9

PrimerR: ACTCTTCTTCTTATTCTCTCTGGTGA Real-time PCR 0.5 0.8 1.5 1.2

Probe: TGCACTCTGTCTTTCCTCACTCCGCT

IpCG00950 (unknown)

PrimerF: ACTGGTCGTGTATTGGGTGATTT Microarray 1.2 1.6 1.6 1.7

PrimerR: GGCTGCACAGCTCCATGC Real-time PCR 1.0 2.1 1.9 1.2

Probe: CCTGGGACACCCTTGCTGTTCCTCTT
